# Unique genetic signature and selection footprints in Dutch population of German Longhaired Pointer dogs

**DOI:** 10.1111/age.13253

**Published:** 2022-08-22

**Authors:** Yun Yu, Langqing Liu, Jack Windig, Mirte Bosse, Martien A. M. Groenen, Richard P. M. A. Crooijmans

**Affiliations:** ^1^ Animal Breeding and Genomics Wageningen University & Research Wageningen The Netherlands; ^2^ Division of Evolutionary Biology, Faculty of Biology Ludwig‐Maximilians‐Universität (LMU) München Planegg‐Martinsried Germany; ^3^ Centre for Genetic Resources the Netherlands Wageningen University & Research Wageningen The Netherlands; ^4^ Amsterdam Institute for Life and Environment (A‐Life) Vrije Universiteit Amsterdam Amsterdam The Netherlands

**Keywords:** genetic relationship, inbreeding, pointer dog, runs of homozygosity, selection signature

## Abstract

The German Longhaired Pointer (GLP) breed is a versatile pointer dog breed. In the current study, we investigated the genetic diversity of these dogs based on SNP array data and compared it to 11 other pointer setter breeds. The results show that GLPs have a relatively low level of inbreeding among these pointer breeds. In addition, with the availability of pedigree information of the GLPs, we demonstrate that the correlation between pedigree‐based inbreeding and genotype‐based inbreeding coefficients was high (*R* = 0.89 and 0.85). By investigating population structure between these 12 pointer setter breeds we showed that GLP is a breed distinct from other pointers and shares common ancestry with a few other pointing breeds. Finally, we identified selection signatures in GLPs using the runs of homozygosity islands method. The most significant runs of homozygosity island was detected on chromosome 30 harboring the genes *RYR3*, *FMN1*, and *GREM1*. The *RYR3* gene plays a role in skeletal muscle contraction while the *FMN1* and *GREM1* genes are involved in limb development. The selection on these three genes could have contributed to the excellent athletic performance of GLPs, which is an extremely important characteristic for this hunting dog.

## INTRODUCTION

The German Longhaired Pointer (GLP) is a type of multipurpose gundog, which is very active and athletic in general. GLP was assigned as a Spaniel type of continental pointer dogs by Fédération Cynologique Internationale (FCI; http://www.fci.be). Pointer dogs use their instinct to point game (quarry etc.) by stopping and aiming its muzzle towards game. There are 36 pointer breeds according to FCI. These pointer dogs have one recognized ancestor, the Old Spanish Pointer, which was probably established in 100–250 BC and is now practically extinct (Parra et al., 2008). The GLP is one of the oldest continental Pointers. The origin of the GLP dog breed is rather complex and not completely resolved. According to historical records, four hunting breeds potentially contributed to the final GLP breed, namely the German ‘Vogel or Habischtshund’ (Quail dog), the ‘Wasserhund’ (Water dog), ‘langhaarige Jagdhunde’ (German Longhair), and the Spanish ‘Wachtelhund’ (Silk dog) (Kern & Tobolik, 1996; Merx & Merx, 1997). However, whether and how much these breeds were used in the final breed formation is unknown. Meanwhile, these breeds are either extinct or rather rare, making a genomic investigation on origin of the GLP difficult. GLPs are versatile in their ability to pointing birds in the field and trace the hunted prey. In 1879 the breed characteristics for the German Longhair pointer were established, with the most important selection trait of detecting the location of shot animals and bringing them back during hunting. Currently, the GLP breed is thought to be closely related to the German Shorthaired Pointer (GSHP) and German Wirehaired Pointer (GWHP) breeds but closest to the Large Munsterlander (LMUN) breed (Schmutz et al., 2002).

Pedigreed dogs have high inbreeding rates (Lewis et al., 2015; Wijnrocx et al., 2016) and extensive use of popular sires is one of main causes for this (Leroy & Baumung, 2011). Traditionally inbreeding is estimated from pedigrees, but nowadays genomics can provide additional information (Doekes et al., 2021). Besides the popularity of GLP in Germany, breeding GLPs is also popular in the Netherlands. The Dutch population of GLP was found to be predisposed to a genetic form of follicular cell thyroid carcinoma (FCC) (Yu et al., 2022). Inbreeding analysis based on both pedigree and SNP array genotype data indicated that inbreeding contributed to the high incidence of the genetic FCC in the population (Yu et al., 2021). The genetic FCC was found mainly in the Dutch population of GLPs in recent years with onset of cancer formation from age 4.5 to 13.5 years (on average 8.63 years). Due to cross breeding using affected or carrier Dutch GLPs, genetic FCC is also becoming a problem in GLPs in other countries.

The aim of this study is to genetically characterize the GLP breed because no report on the genetic characterization of the GLP breed using genomic data is currently available. In this study, we investigated the genetic relationship between Dutch GLPs and several other pointer setter breeds by looking into the population structure. The pointer setter breeds were defined based on phylogenetic clustering (Parker et al., 2017). To obtain insight in inbreeding in GLPs, we also compared the inbreeding level between GLP and these pointer setter breeds. Within‐breed genetic diversity of these pointer setter breeds was also assessed using runs of homozygosity (ROH) and linkage disequilibrium decay. These results could be valuable to guide breeding programs of GLP. Finally, we investigated selection signatures in GLPs using the ROH islands method. Those selected genomic regions may underlie specific characteristics of GLPs.

## MATERIALS AND METHODS

### Study population

We genotyped 58 Dutch GLPs in a previous study with either the 170K or 230K canine SNP array (Yu et al., 2021). Among the 58 dogs, there were some full siblings. We therefore randomly selected only one dog from each full sibling cluster, which resulted in 37 GLPs to be included in this study. All these GLPs were born in the Netherlands between 1997 and 2007, and therefore only representing the Dutch population of the GLP breed. Additionally, a publicly available dataset was obtained where 1346 dogs from 161 breeds were genotyped with a 150K SNP array (Parker et al., 2017). All these dogs were used in the phylogenetic analysis to determine the genetic relationship between GLP and other dog breeds, while for other analyses based on genotype data in this study, only dogs classified as pointer setter were used (Table 1), including 10 Brittany dogs (BRIT), 10 English Setters (ESET), 10 Gordon Setters (GORD), 10 GSHP, two GWHP, nine Irish Setters (ISET), three LMUN, two Spinone Italiano dogs (SPIN), seven Vizsla dogs (VIZS), 10 Weimaraner dogs (WEIM), and six Wirehaired Pointing Griffon dogs (WHPG). According to classification of FCI, BRIT is a Scent hound, and ESET, GORD, and ISET are Setters. Furthermore, we utilized whole genome sequences (WGS) of 22 GLPs that were generated in our previous study to validate and finemap the signatures of selection (Yu et al., 2021). The mapping and variant calling have been described in our previous study (Yu et al., 2021).

**TABLE 1:** Pointer setter dogs included in this study.

Dog breed	Abbreviation	Origin country	FCI^a^ classification	Number of dogs^b^
German Longhaired Pointer	GLP	Germany	Pointer dog	37
Brittany	BRIT	France	Scent hound	10
English Setter	ESET	Great Britain	Setter	10
Gordon Setter	GORD	Great Britain	Setter	10
German Shorthaired Pointer	GSHP	Germany	Pointer dog	10
German Wirehaired Pointer	GWHP	Germany	Pointer dog	2
Irish Setter	ISET	Ireland	Setter	9
Large Munsterlander	LMUN	Germany	Pointer dog	3
Spinone Italiano	SPIN	Italy	Pointer dog	2
Vizsla	VIZS	Hungary	Pointer dog	7
Weimaraner	WEIM	Germany	Pointer dog	10
Wirehaired Pointing Griffon	WHPG	Netherlands/Germany/France	Pointer dog	6

^a^ Fédération Cynologique Internationale.

^b^ GLPs were genotyped with either 170K or 230K canine SNP array in our previous study (Yu et al., 2021). The genotype data of the rest dogs was collected from the study of Parker et al. (2017).

### Pedigree analysis

A pedigree consisting of 58 533 GLPs worldwide was provided by the GLP breed association. Some errors were detected in pedigree and corrections were made. Impossible birth years (e.g., 19981 or 201) were set to unknown. Date of birth of dogs whose parents were born after their birth was also set to unknown. A pedigree loop involving a GLP was detected. To correct this, the mother of that GLP was set to unknown. The Retriever program (Windig & Hulsegge, 2021) was used to perform pedigree‐based analyses, such as pedigree completeness assessment (complete generation equivalent), contribution of top sires, generation interval, and litter size. The complete generation equivalent was estimated as sum of the proportions of known ancestors of an individual over all traced generations. Meanwhile, pedigree completeness for each country, defined as the proportion of known ancestors in each generation within a country, was also assessed using optiSel package (Wellmann, 2019). Moreover, pedigree‐based inbreeding coefficient (*F*_ped_) was estimated using the CFC (Coancestry, inbreeding [*F*] and Contribution) program (Sargolzaei et al., 2006). Popular sires were also identified and a popular sire was defined as a male dog that sired at least 32 offspring, corresponding to five litters based on observed average litter size.

### Genotype data

The overlap between the three (150, 170, and 230K) SNP genotype sets was determined based on exact location (chromosome + position) of markers and used to merge all genotype data into a single SNP genotype dataset. plink program (v1.9) (Purcell et al., 2007) was used to perform quality control with following criteria: minor allele frequency (‐‐maf) >0.05; missing genotype per individual (‐‐mind) <0.05; missing call rate per marker (‐‐geno) <0.05, Hardy–Weinberg equilibrium exact test *p*‐value (‐‐hwe) >0.000001.

### Genetic relationship between breeds

To determine the relationship between the pointer setter breeds, a principal component analysis (PCA) was performed using plink (v1.9) and r (v4.0.3). Firstly, genetic distance between dogs was calculated using ‘‐‐distance‐matrix’ in plink (v1.9). Then, classic multidimensional scaling of the distance matrix was performed using ‘cmdscale’ function in R and the first and second component were plotted using R. To investigate the genetic similarities between all dog breeds (GLP + 161 other dog breeds), a phylogenetic tree was constructed. Firstly, the pairwise distance between dogs was estimated using plink with command ‘‐‐distance 1‐ibs’. The ape package (Paradis et al., 2004) was then used to construct a neighbor‐joining phylogenetic tree from the distance matrix using ‘nj’ function in default settings. The phylogenetic tree was visualized and modified using the figtree program (v1.4.4; http://tree.bio.ed.ac.uk/software/figtree/).

### Inbreeding estimation

Besides *F*_ped_, the inbreeding coefficient was also estimated based on SNP array data and compared between the pointer breeds. Homozygous genotype‐based inbreeding coefficient (*F*_HOM_) was estimated by plink with command ‘‐‐het’, which is based on expected and observed autosomal homozygous genotypes. ROH across the genome were detected using plink (v1.9) through a sliding window approach. ROH were defined according to the following criteria: (i) the minimum count of SNPs in a sliding window was 50; (ii) the minimum ROH length was set to 1 Mb; (iii) the maximum inverse density was 50 kb per SNP; (iv) To avoid the effects of low SNP density region, the maximum gap length between consecutive SNPs was 1 Mb; (v) The minimum hit rate of all scanning windows containing the SNP was set to 0.05; (vi) at most one heterozygous call allowed per scanning window; (vii) at most five missing calls allowed per scanning window. Total length of ROH for each dog was plotted by breeds. *F*_ROH_ was estimated by total length of ROH segments on auto‐chromosomes divided by total length of auto‐chromosomes (2200 Mb). To eliminate the possibility that SNP chip ascertainment bias results in false positives and to validate ROH hotspots, ROH were also identified based on WGS data in plink according to the following criteria: (i) the minimum count of SNPs in a sliding window was 50; (ii) the minimum ROH length was set to 500 kb; (iii) the maximum inverse density was 30 kb per SNP; (iv) to avoid the effects of low SNP density region, the maximum gap length between consecutive SNPs was 1 Mb; (v) the minimum hit rate of all scanning windows containing the SNP was set to 0.05; (vi) at most five heterozygous call allowed per scanning window to account for false heterozygous calls from WGS data; and (vii) at most three missing calls allowed per scanning window.

### Population structure

To investigate the genetic relationship between these pointer breeds, the admixture (v1.3.0) program (Alexander et al., 2009) was used to estimate the population structure base on genotype data with inferred cluster (*k* value) from 2 to 12. The best *k* value was then determined when a smallest cross‐validation error was achieved from the observed data.

### Extent of linkage disequilibrium

To eliminate the potential bias introduced by a larger sample size of GLP, we randomly sampled 10 GLPs for linkage disequilibrium (LD) decay analysis. The LD was measured using *r* ^2^ between pairs of markers. poplddecay (Zhang et al., 2019) was used to calculate the LD decay for sub‐populations identified in GLPs and each pointer breed with a maximum distance of 2000 kb between markers. Next to this, the accompanying Plot_MutiPop.pl perl script was used to plot the LD decay curve with parameters: ‐bin1 100; ‐bin2 3000; ‐break 2000; ‐method MeanBin. Due to a small sample size for GWHP, SPIN, and LMUN, these three breeds were not included in LD decay analysis.

### Detection of selection signature

We identified SNPs with selection signatures in GLPs by detecting ROH islands across all autosomal chromosomes based on both SNP array and WGS data separately (see ROH analysis in Inbreeding estimation section). We plotted the percentage of dogs with the SNP in a ROH against the chromosome location. The ROH island was defined to be genomic region where frequency of presence of SNPs in a ROH is in the top 1% of distribution of that frequency among all GLPs. Genes within the overlapping ROH islands identified based on SNP array and WGS data were extracted and a gene set enrichment analysis was performed using clusterprofiler package (Yu et al., 2012).

## RESULTS

### Pedigree based analysis

**FIGURE 1:**
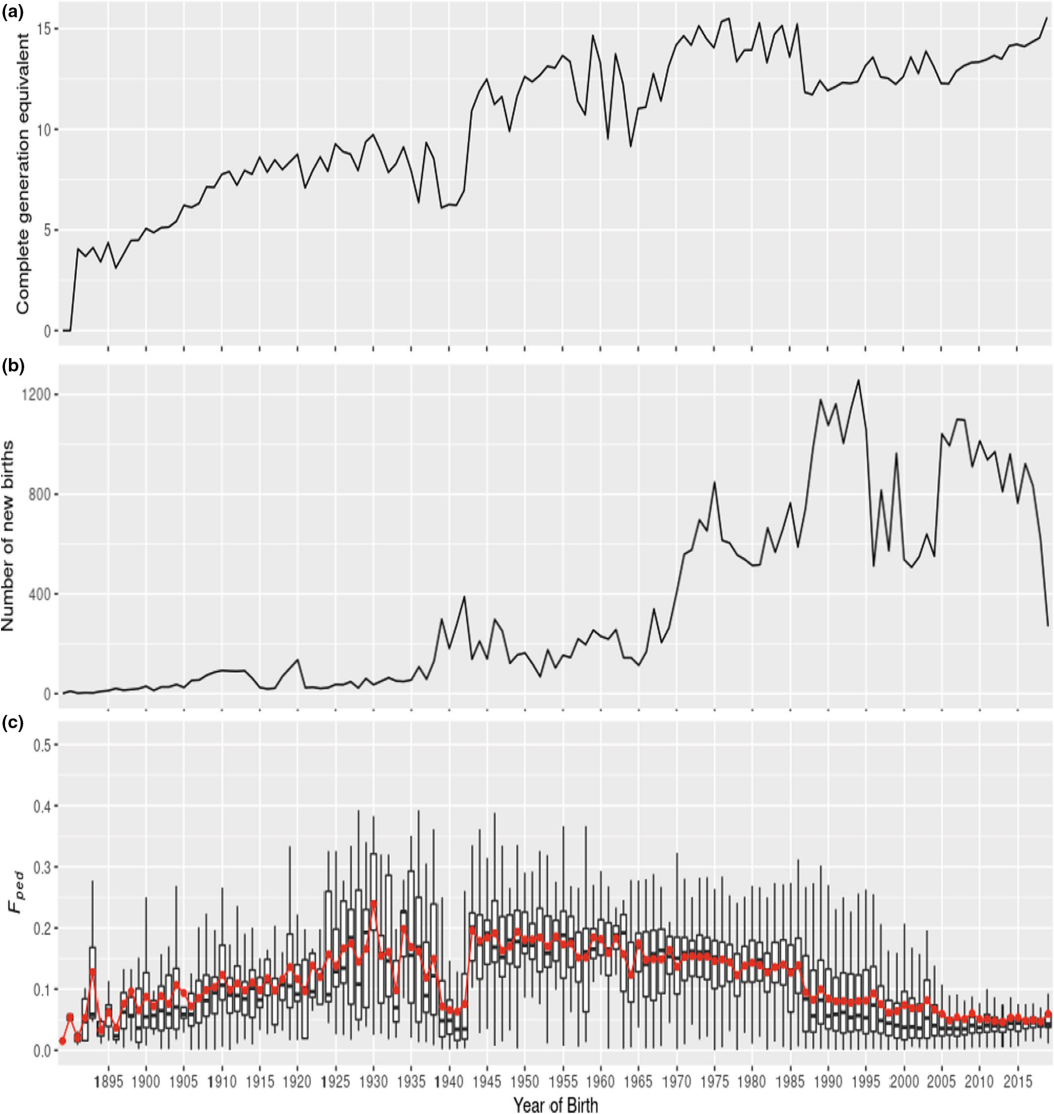
(a) Average generation equivalent of German Longhaired Pointers born each year between 1882 and 2019. (b) Number of newborn puppies per year between 1882 and 2019. (c) Pedigree‐based inbreeding (*F*_ped_) of German Longhaired Pointers born each year from 1882 to 2019. Red dots indicate the average values of *F*_ped_.

Pedigree information from 58 533 GLPs worldwide was available where the ancestor can be traced back to 1876. Yearly average complete generation equivalent of dogs born after 1990 was more than 10. However, most dogs, especially dogs born before 2013, have a complete generation equivalent of <5. Meanwhile, average complete generation equivalent has not gone up since 1960s (Figure 1a). These suggest incompleteness of the pedigree. The average yearly number of puppies born worldwide between 1990 and 2019 was 945 (ranging from 609 to 1420; Figure 1b). Average litter size was 6.1 puppies across cohorts between 1990 and 2019. Average generation interval was 4.93 years.

Before 1938, *F*_ped_ increased steadily (Figure 1b). From 1943 to 1986, *F*_ped_ slightly and continuously decreased from 0.196 to 0.139. In 1987, there was a sharp decrease of *F*_ped_ and complete generation equivalents, implying that probably some new dogs from other countries were used in the breeding in that year. After that, overall, *F*_ped_ kept decreasing slowly (Figure 1c). Most GLPs were born in Germany (18 077 dogs) and the Netherlands (16 669 dogs). GLPs born in the Netherlands had higher inbreeding levels compared to GLPs in Germany (0.137 > 0.048, *p* < 2.2e‐16, Wilcoxon rank sum test). However, the pedigree completeness of GLPs born in Germany is lower than that of GLPs born in the Netherlands (Figures S1–S12). This might result in lower estimated inbreeding of GLPs born in Germany than in the Netherlands. Moreover, relationship between GLPs across countries (Germany and the Netherlands, 0.024) is more distant than that within country (within the Netherlands: 0.057, within Germany: 0.048; Figure S2).

There were 4177 sires in the GLP pedigree, of which 471 males sired more than 32 puppies each. These 471 popular sires produced 29 519 offspring in total, which sum up to 50% of the GLPs included in the pedigree. Between 1990 and 2019, the contribution of top 10 popular sires ranged from 18.1% to 47.9% per year, with an average of 28.9%. This suggests a strong sire effect among GLPs. Of the male pups, 9.7% produced offspring later in life whereas 17.9% of the female pups produced offspring. The most popular sire was born in 1984 and produced 292 puppies. The most popular dam was born in 1964 and gave birth to 67 puppies.

### Genetic distance between pointer dog breeds

**FIGURE 2:**
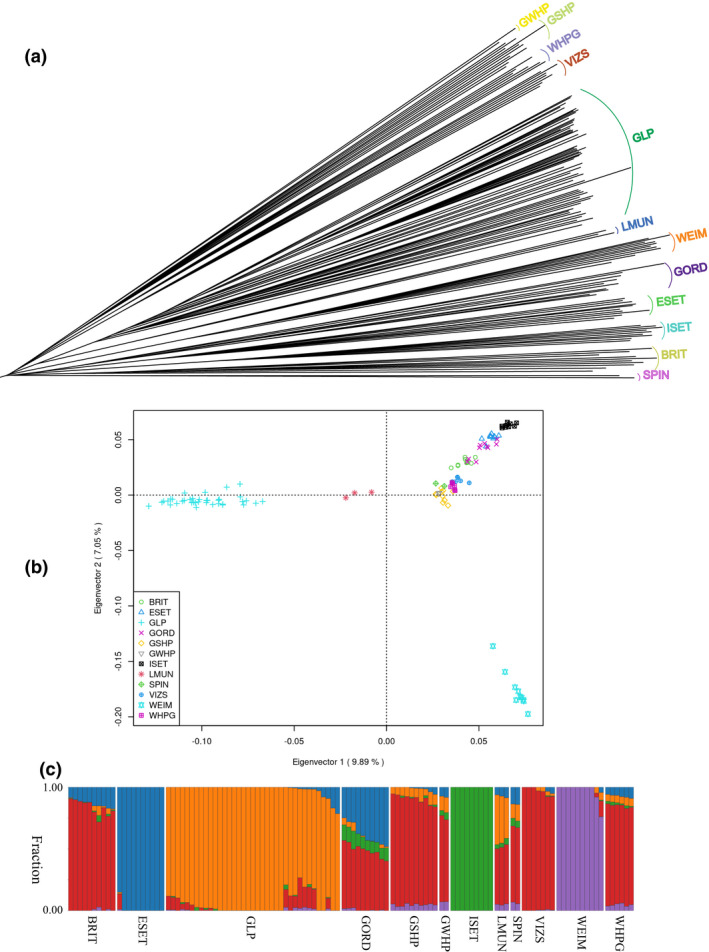
(a) Subclade of 12 pointer setter breeds on the neighbor‐joining phylogenetic tree constructed based on genotype data of 1386 dogs from 162 breeds. (b) Principal component analysis plot of the 12 pointer setter breeds. (c) Population structure between the 12 pointer setter breeds estimated by admixture with inferred cluster of 5. BRIT, Brittany; ESET, English Setter; GLP, German Longhaired Pointer; GORD, Gordon Setter; GSHP, German Shorthaired Pointer; GWHP, German Wirehaired Pointer; ISET, Irish Setter; LMUN, Large Munsterlander; SPIN, Spinone Italiano; VIZS, Vizsla; WEIM, Weimaraner; WHPG, Wirehaired Pointing Griffon

From the three SNP sets (150, 170, and 230K) 146 324 common SNP markers were selected and finally 126 144 SNPs remained after quality control. To determine the phylogenetic relationship between GLP and 161 other dog breeds, a neighbor‐joining tree was constructed (Figure S3). The GLPs, as expected, are located on the clade of pointer setters together with the other pointer setter breeds (highlighted in red in Figure 2a). Moreover, all German pointer breeds were within a clade, although VIZS from Hungary also appeared within this clade. VIZS is on the same sub‐clade as GSHP, GWHP, and WHPG (Figure 2a). To investigate the genetic distance between the 12 pointer setter dog breeds, a PCA analysis was performed (Figure 2b). The PCA result shows that GLPs are well separated from other pointer setter breeds, and intra‐population difference among the GLPs was also seen. The first component could differentiate GLP and LMUN from the other pointer setter breeds. The LMUN breed is the closest breed to the GLP in the PCA plot and also in the phylogenetic tree, which is in agreement with our knowledge about the breeding history of these two breeds. The second component differentiates the WEIM breed from the other pointer breeds.

### Population structure of pointer setter dogs

To characterize the population structure and admixture patterns among these pointer setter breeds, admixture was run from *k* = 2 to *k* = 12 (Figure S4). According to the cross validation, the cluster number that describes the study population the best was *k* = 5 (Figure S5). At *k* = 2, the first breed to differentiate from the others are the GLPs. At *k* = 3, both WEIM and ISET are separated from other dogs. At *k* = 5, five distinct breeds are identified: ESET, GLP, ISET, VIZS, and WEIM (Figure 2c). Genetic components of these five breeds are mixed in other breeds.

The genetic components identified mainly in GLPs are also detected in some of the other breeds (BRIT, GORD, GSHP, GWHP, LMUN, SPIN, VIZS, WEIM, and WHPG), but are not present in English setters and Irish setters. This suggests that those 10 breeds (except for ESET and ISET) may have shared ancestry and GLP may resemble most the ancestral breed. Among all these breeds, LMUN genetically share most with GLPs, which was consistent with the PCA result. According to the breeding history, LMUN was separated in 1909 from GLP according to a difference in coat color (black color). From *k* = 2 to *k* = 12, we did not identify a significant proportion of genetic component coming from another breed admixed into GLPs, thus these 11 pointer setter breeds are unlikely to have served as an ancestral breed used in the development of the GLP breed.

### Relatively low inbreeding level of GLP among pointers

**FIGURE 3:**
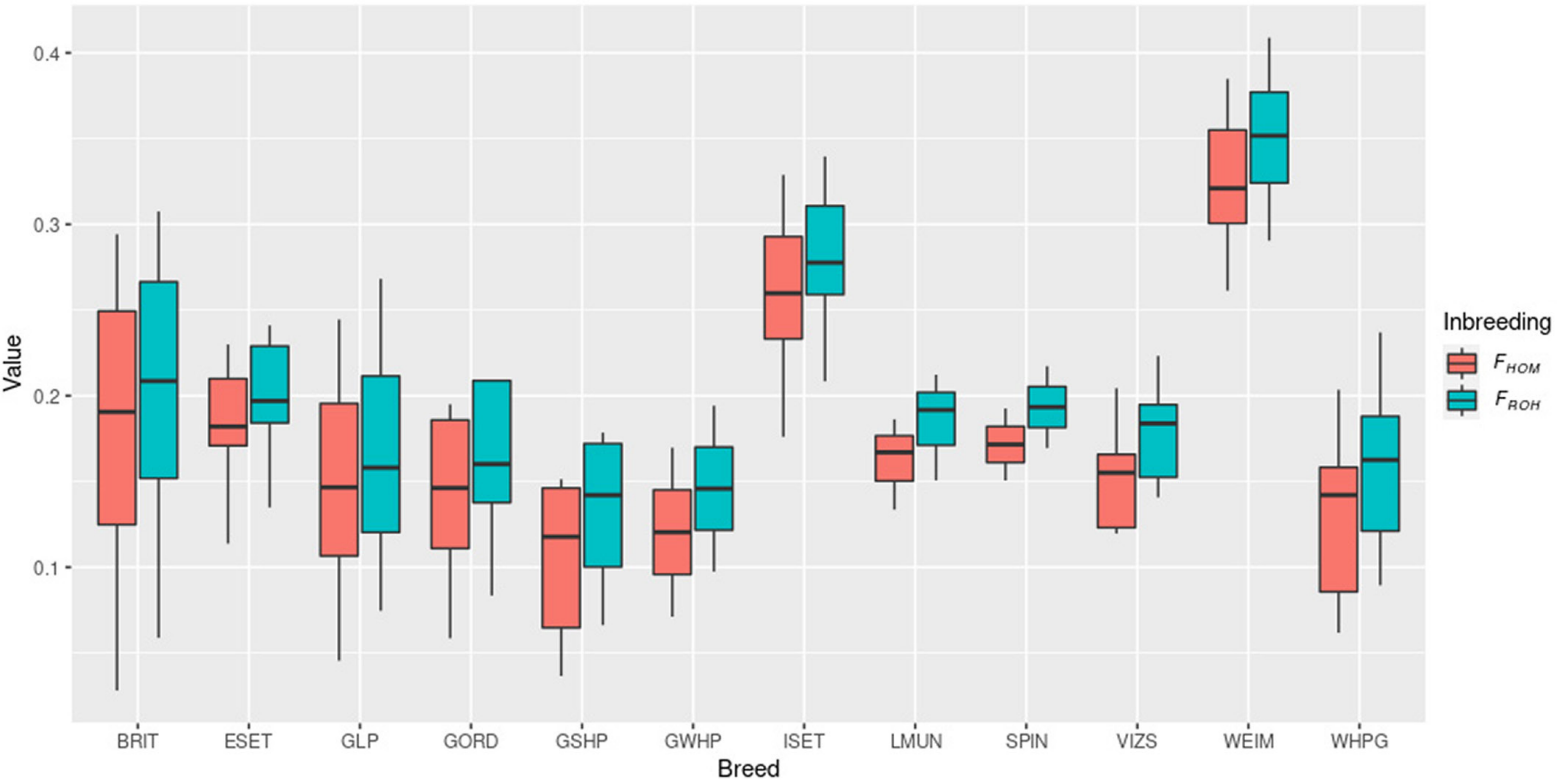
Boxplot of *F*_ROH_ and *F*_HOM_ for all the dogs grouped by breed. BRIT, Brittany; ESET, English Setter; GLP, German Longhaired Pointer; GORD, Gordon Setter; GSHP, German Shorthaired Pointer; GWHP, German Wirehaired Pointer; ISET, Irish Setter; LMUN, Large Munsterlander; SPIN, Spinone Italiano; VIZS, Vizsla; WEIM, Weimaraner; WHPG, Wirehaired Pointing Griffon

To assess the inbreeding level of GLPs, we estimated the *F*_ROH_ and *F*_HOM_ of each dog and compared the results between the pointer setter breeds. Among the 12 pointer setter breeds, GLPs have relatively low average inbreeding level based on both *F*_ROH_ and *F*_HOM_ (Figure 3). The average *F*_ROH_ of GLPs is 0.16. Compared to its cousin breeds, GSHP and GWHP, GLP has a slightly higher inbreeding level. Among all these pointer setter breeds, ISET and WEIM have the highest inbreeding level. Moreover, three inbreeding parameters, *F*_ped_, *F*_HOM_ and *F*_ROH_ of the 37 GLPs, have high concordance (Figure S6), and *F*_ROH_ and *F*_HOM_ estimated from genotype data had the highest correlation coefficient (0.99).

### LD decay

**FIGURE 4:**
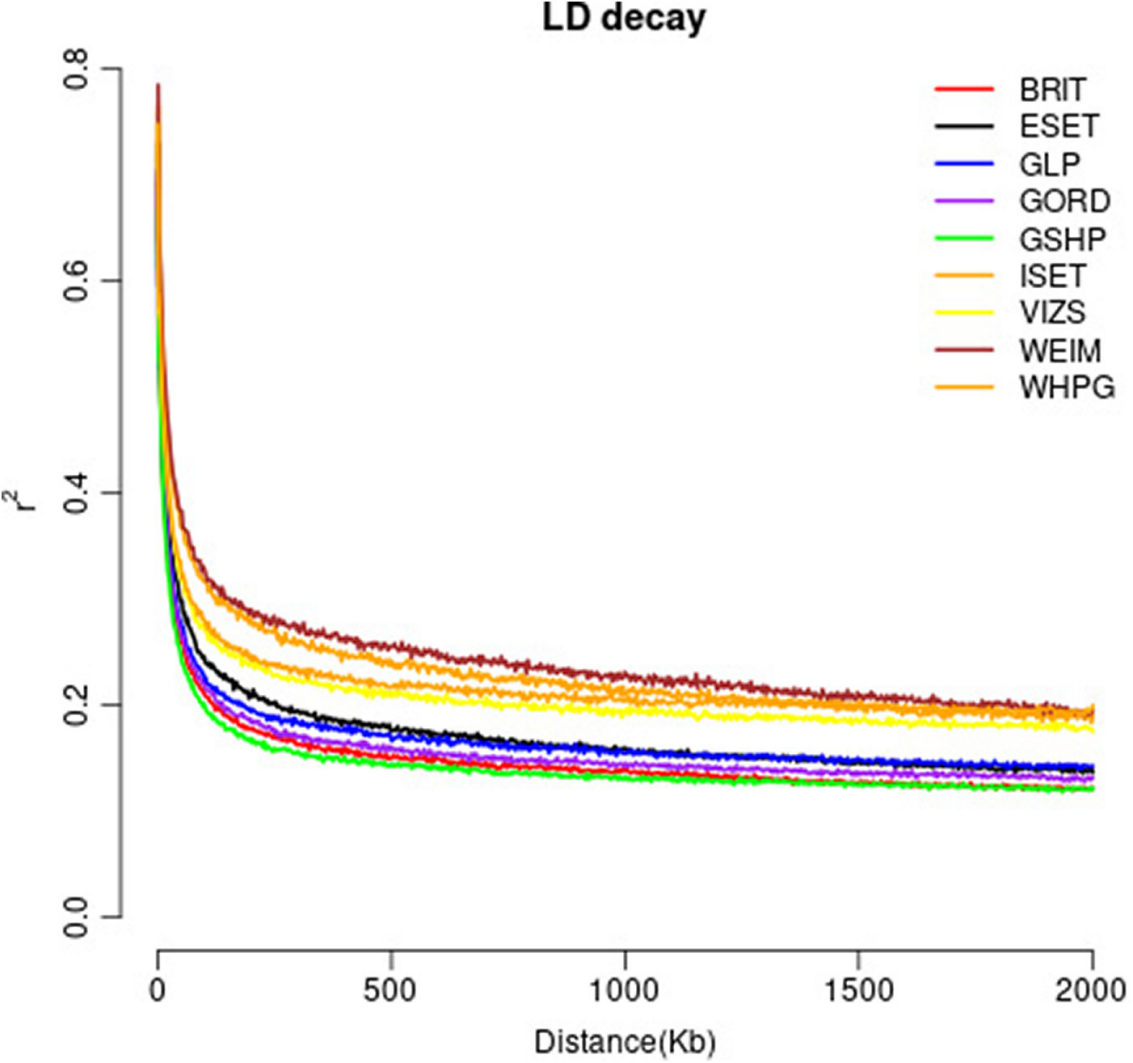
Linkage disequilibrium (LD) decay estimated for nine pointer breeds. BRIT, Brittany; ESET, English Setter; GLP, German Longhaired Pointer; GORD, Gordon Setter; GSHP, German Shorthaired Pointer; ISET, Irish Setter; VIZS, Vizsla; WEIM, Weimaraner; WHPG, Wirehaired Pointing Griffon

To reduce the bias on estimates of LD decay because of unbalanced sample size, we randomly sampled 10 GLPs for LD decay analysis. Meanwhile, SPIN, LMUN, and GWHP were not included in this study because of a small sample size (two or three dogs per breed). The extent of LD, in general, corresponds well to the inbreeding level of the breed (Figure 4). ISET and WEIM have longest extent of LD where they also have highest inbreeding. GSHP, GORD, and GLP have lower inbreeding level where they also have shorter extent of LD.

### Genomic distribution of ROH

**FIGURE 5:**
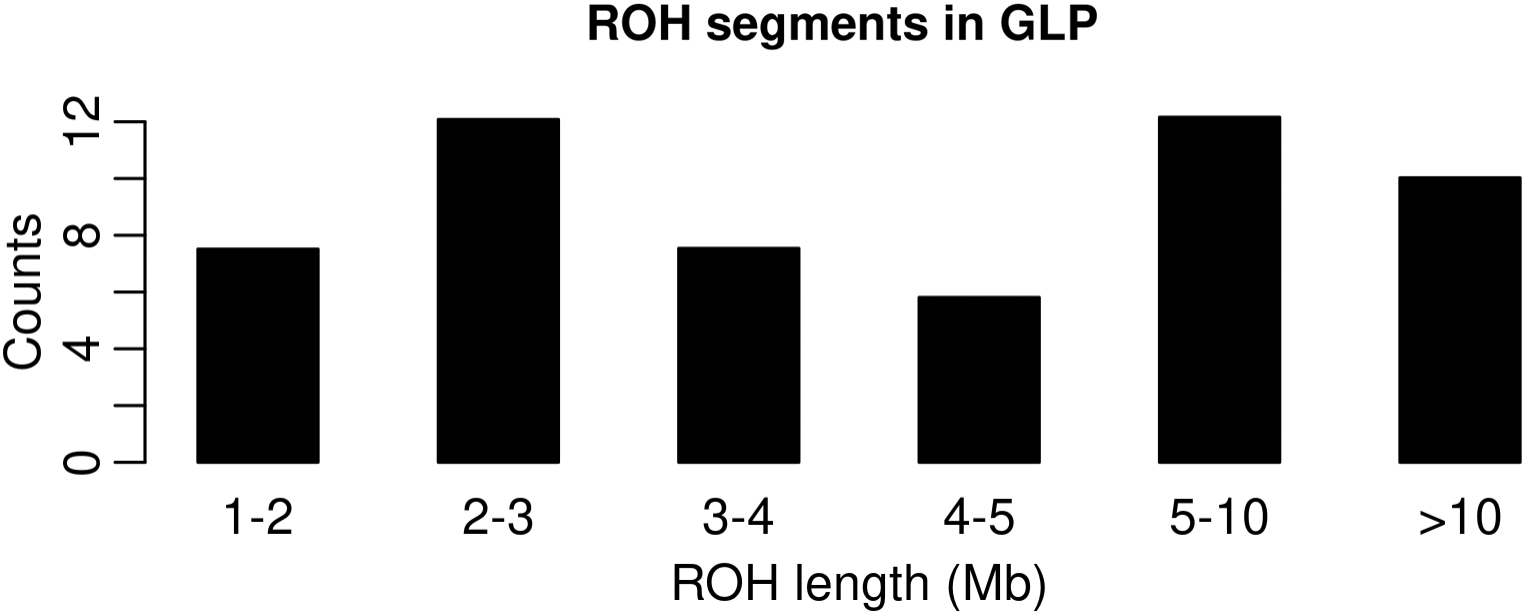
Length distribution of runs of homozygosity (ROH) per German Longhaired Pointer. *Counts* in the vertical axis means count of ROH segments in different length ranges.

In total, 2040 ROH with an average length of 6.57 Mb (ranging from 1.29 to 64.72 Mb) were identified in the 37 GLPs based on the SNP array data. ROH are divided into six groups according to size (Figure 5), where 18% of them are longer than 10 Mb. Short ROH reflect ancestral inbreeding, while longer ROH reflect more recent inbreeding. Length distribution of ROH indicates that both ancient and recent inbreeding events have affected genomic diversity in current GLPs.

**FIGURE 6:**
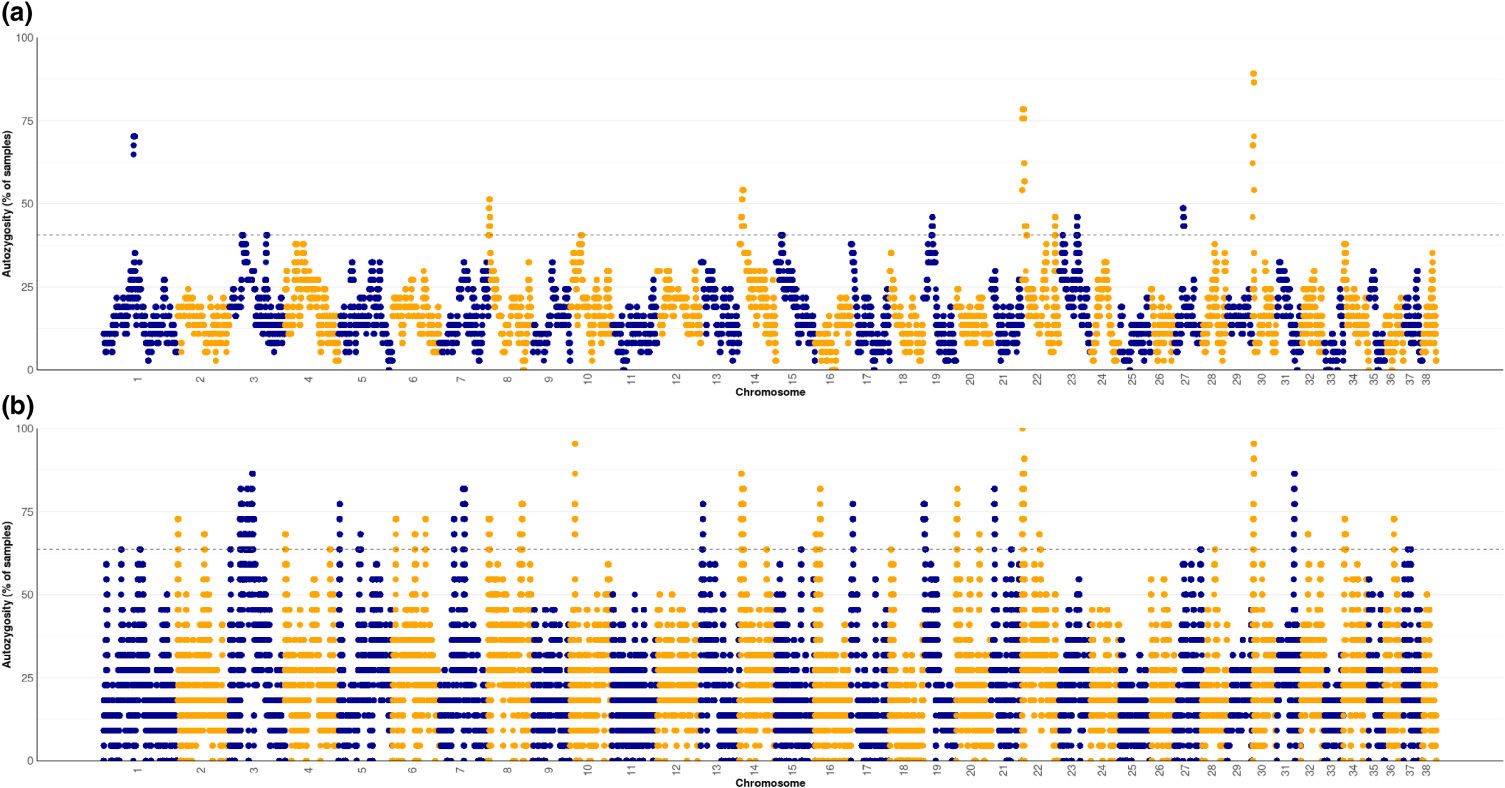
Runs of homozygosity islands identified on autosomes based on SNP array (a) and whole genome sequence (b) data of German Longhaired Pointers. Dashed line indicated the threshold of top 1% of empirical distribution of autozygosity.

**FIGURE 7:**
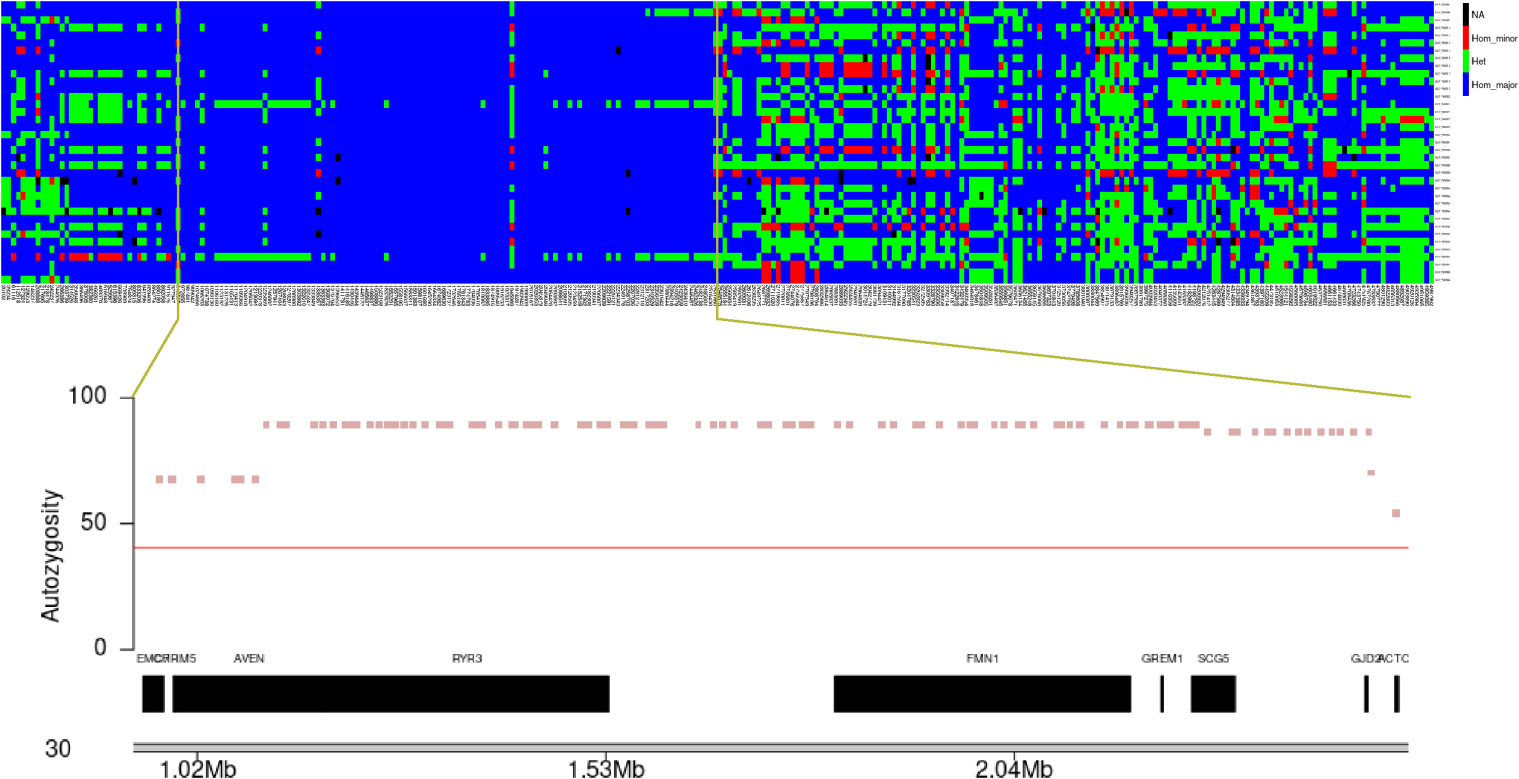
Genotypes of the variants between 0 and 5 Mb on chromosome 30 in German Longhaired Pointers. Colors denote: Homozygous for major allele (allele), heterozygous (green), homozygous for minor allele (red), and missing genotype (black). The plot below the genotype panel show the genes within the zoomed region. The *x*‐axis shows the coordinates on chromosome 30 and *y*‐axis shows the percentage of dogs with the SNP in the runs of homozygosity.

Runs of homozygosity islands among these GLPs are identified based on either SNP array data (Figure 6a) or WGS data (Figure 6b) separately. Overlapping ROH islands between datasets were identified on chromosomes 8, 14, 22, and 30. These common ROH with low genetic diversity imply genomic signatures of selection. Genes in those overlapping ROH islands data were extracted and a gene set enrichment analysis was performed. However, no enriched GO term or KEGG pathway was identified. The 1.6‐Mb ROH island on chr30 indicates homozygosity for nearly all GLPs, and contains seven genes, of which three are very interesting because of their central location in the ROH segment and their function (Figure 7). The *RYR3* (ryanodine receptor type 3) gene is involved in skeletal muscle contraction by releasing calcium from the sarcoplasmic reticulum followed by depolarization of T‐tubules (Lynch et al., 2013), the *FMN1* (Formin 1) gene was reported to associate with limb deformity in mouse (Zhou et al., 2009) and the *GREM1* (Gremlin 1) gene is also known to play a role in limb outgrowth and development in mammals (Khokha et al., 2003).

**FIGURE 8:**
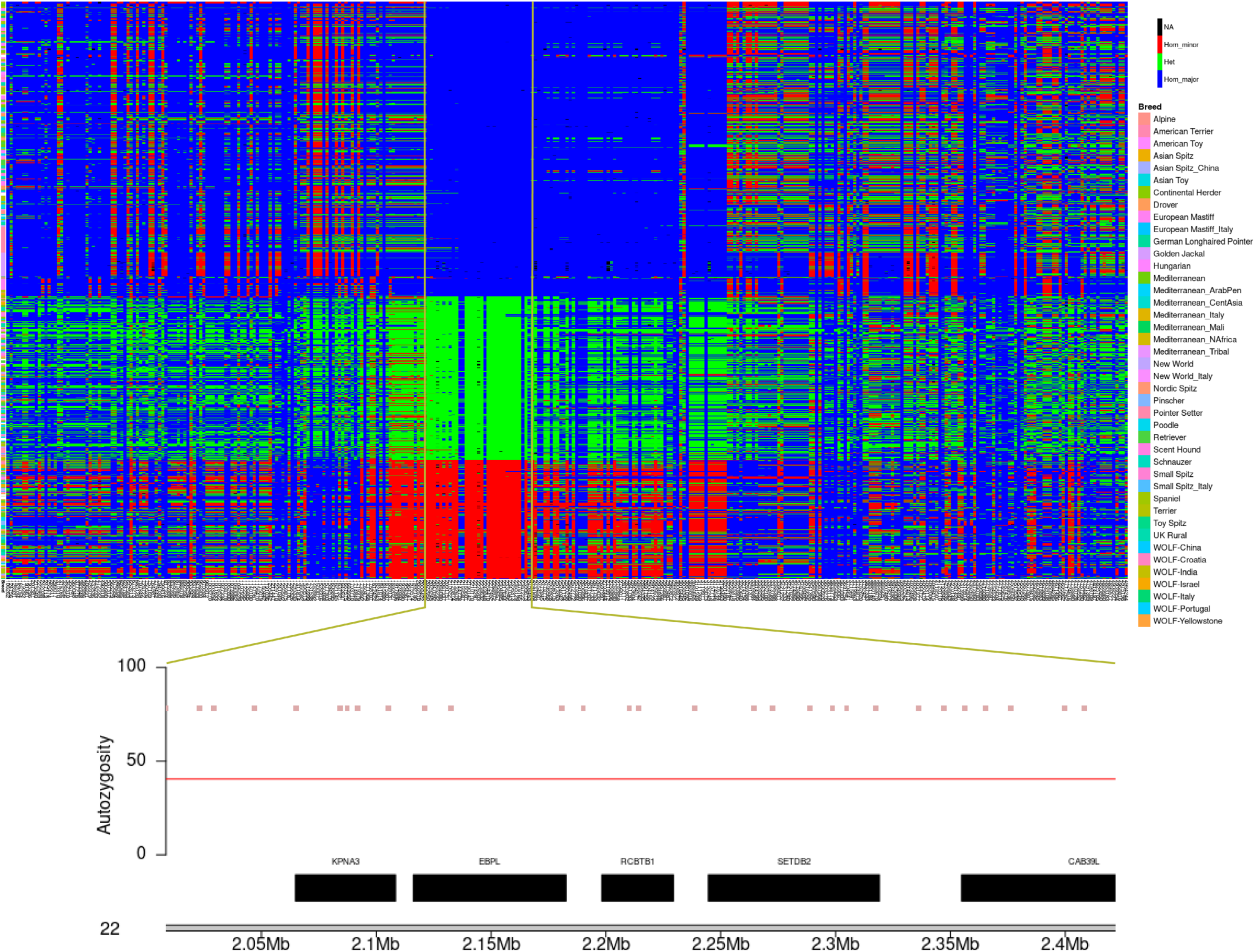
Upper panel shows genotypes of the variants between 0 and 5 Mb on chromosome 22 in 37 German Longhaired Pointers and 1346 other dogs from 161 different breeds. Colors denote: Homozygous for major allele (allele), heterozygous (green), homozygous for minor allele (red), and missing genotype (black). Panel below shows the genes in the zoomed runs of homozygosity region. The *x*‐axis shows the coordinates on chromosome 22 and *y*‐axis shows the percentage of dogs with the SNP in the runs of homozygosity.

Moreover, a ROH island on chr22 was also identified and the corresponding region is an outlier not only in GLPs, but also in many other dog breeds (Figure 8). Interestingly, an alternative haplotype also occurs at high frequency in this region, hinting at low recombination. Five genes are located within this region: *KPNA3* (karyopherin subunit α3), *EBPL* (emopamil binding protein like), *RCBTB1* (RCC1 and BTB domain‐containing protein), *SETDB2* (SET domain bifurcated histone lysine methyltransferase 2), and *CAB39L* (calcium binding protein 39 like).

We also investigated ROH islands identified on other chromosomes (chromosome 8, 14, 27). These regions are less homogeneous among GLPs (Figures S7–S9), implying that these regions are not completely fixed in these GLPs and were therefore not further investigated.

## DISCUSSION

In this study, we investigated the phylogenetic relationship and population structure of GLP and 11 other pointer setters. The GLP belongs to the pointer setter cluster on the phylogenetic tree as expected. The phylogenetic tree and PCA analysis show that LMUN is the breed closest to GLP, followed by the GWHP, GSHP, WHPG, and VIZS. This corresponds well to the recorded breeding history of these dog breeds.

We estimated *F*_ROH_ and *F*_HOM_ and compared them between 12 pointer setter breeds included in this study. The average inbreeding of three breeds, GLP, GSHP, and GWHP, was lower compared to the other pointer breeds, while a big variation within the breeds was also observed. One thing to note is that the relatively low inbreeding of GLP, GSHP, and GWHP among 12 pointer setter breeds included in the current study does not indicate that these three breeds have absolutely low inbreeding and do not suffer from adverse effects of inbreeding. It is known that pedigree dogs have high inbreeding rates in general due to two bottlenecks in the history (Lewis et al., 2015; Wijnrocx et al., 2016; Zhang et al., 2020). A subpopulation or country effect on the inbreeding estimation cannot be excluded. For instance, we observed a higher inbreeding level in GLPs born in the Netherlands than those in Germany, although this could also be due to unreliable pedigree. Lower inbreeding would indicate fewer inbreeding related health issues as we know that inbreeding can cause inbreeding depression (Ujvari et al., 1875). Dutch GLPs were found to be predisposed to FCC. Although the inbreeding level in Dutch GLPs is relatively low in comparison to other 11 pointer setter breeds according to the current study, inbreeding level in certain subpopulations of GLPs is high and this probably contributes to the high incidence of follicular cell thyroid carcinoma. (Yu et al., 2021, 2022). To eradicate genetic defects from GLPs, inbreeding control is essential. WEIM and ISET have the highest inbreeding level among these pointer setter dogs. These two breeds have been reported to be susceptible to certain diseases, such as canine hypertrophic osteodystrophy (Ferguson & Sandu, 2012). Inbreeding based on pedigree data was determined for the GLPs. There are four GLPs for which estimated *F*_ROH_ and *F*_HOM_ are much higher than *F*_ped_ (Figure S6). This is likely to be caused by incomplete pedigree of these dogs (Figure S11). Meanwhile, pedigree errors could also be an explanation of divergence between *F*_ped_ and genotype‐based inbreeding. Pedigree based inbreeding has a lower correlation with *F*_ROH_ and *F*_HOM_ than the correlation between *F*_ROH_ and *F*_HOM_. Pedigree based inbreeding was less accurate because of incorrect or incomplete records in the pedigree. Also, pedigree based inbreeding estimation is not able to take into account the various stochastic recombination events that occurred during meiosis.

Ancestral breeds used in the development of GLP were not detected through the population structure analysis. In addition, the breeding history of GLP is not completely clear. We thus are not able to disentangle the proportion of genetic component of ancestry in current GLPs. According to the admixture result, with *k* values from 2 to 12, GLP represents a unique breed, without or with very little genetic component from other pointer breeds included in the study. This suggests that the GLP breed might resemble the shared breed ancestry of the continental pointer breeds. While other pointer setter breeds are more mixed with some other pointer setter breeds. At *k* = 5, the genetic component of VIZS is largely seen in many other pointer setter breeds, including GSHP, WHPG, SPIN, GORD, LMNU, and BRIT. It is also seen in some GLPs. It is known that VIZS was used in the development of other pointer breeds, most notably the WEIM and GSHP (Boggs & Boggs, 2000). Our result confirmed this.

The selection signatures in GLPs were identified by exploring the ROH islands based on SNP array data. The selected genomic regions cover several genes that may underlie characteristics that are specific or important to GLP dogs. With the ROH islands detection method, we identified several regions in the genome of GLP characterized by a loss of genetic diversity, implying selection signatures, based on both SNP array and WGS data. The ROH island identified on chromosome 30 contains three genes of interest. One of these, *RYR3*, plays a role in skeletal muscle contraction, which mediates the mobilization of stored Ca^2+^ in cardiac and skeletal muscle to initiate muscle contraction (Lynch et al., 2013). The *RYR3* gene was identified as a candidate gene in selection sweep analysis of hunting dogs (Kim et al., 2018). This gene not only was under selection in GLPs, but also in many other hunting dog breeds. The other two genes in this region on chromosome 30 are *FMN1* and *GREM1. GREM1* is involved in limb development and growth (Khokha et al., 2003). *FMN1* and *GREM1* share the same cis‐regulatory landscape and deletion of a ~180‐kb genomic region overlapping the grem‐fmn1 topologically associating domain disrupts *GREM1* expression in limb buds (Malkmus et al., 2021). Pointer dogs run faster and are more athletic than many other dogs. The selection on these three genes can be linked to breeding practice of the GLPs. An especially muscular loin was included in GLP's breed standard. The selection on *RYR3* may contribute the muscular characteristic of the GLPs. Good legs are important for a hunting dog to support them searching and tracking in the field, water, and forest for long periods. GLP breeders have stringent breed standards about limbs written in the breed standard of GLP, such as shoulder, elbow, carpus, feet, and general appearance of forequarters and hindquarters. The selection on *FMN1* and *GREM1* may contribute to those as muscle and legs are important for hunting performance of a hunting dog.

The selection signature identified on chromosome 22 (from position 2 008 422 to 2 421 940) was also reported in previous studies. Akkad et al. (2015) indicated that one particular gene in the region, *SETDB2*, could contribute to the pointing behavior, because this selection signal was detected in both pointer breeds (LMUN and WEIM), and not in herding dogs (Berger des Pyrenees and Schapendoes). However, our analysis does not support their conclusion. According to our analysis, this selection signal is present not only in pointer breeds, but also in many other breeds. Approximately 51% of all dogs (GLPs and 1346 other dogs) capture the same haplotype detected in GLPs. Especially, we found that some terrier and retriever dog breeds—Bedlington Terrier, Border Terrier, Newfoundland, Irish Water Spaniel, Scottish Terrier, Soft Coated Wheaten Terrier, and Norfolk Terrier—carry the same haplotype as GLP (Figure S10). Therefore, we conclude that the selection signal is not related to specific pointing behavior. This ROH region characterizes with a low recombination and therefore is more sensitive to drift effects, leading to the potential of fixation of haplotypes in these inbred breeds without the need for strong selection for a gene in that region. In humans, *SETDB2* associates with left–right axis differentiation (Ocklenburg et al., 2016). It is unknown if this association with this gene is also the case in dogs. Moreover, there are other genes located within this ROH region on chromosome 22, such as *KPNA3*, *EBPL*, *RCBTB1*, and *CAB39L*. These genes have diverse functions and are involved in different pathways (e.g. NLS‐bearing protein import into nucleus, sterol metabolic process, cell cycle, intracellular signal transduction). *KPNA3* mediates nuclear import (Hu et al., 2019), *EBPL* still has unclear function (Moebius et al., 2003), *RCBTB1* may play a role in angiogenesis (Wu et al., 2016), and *CAB39L* plays a role in the regulation food intake in chicken (Yuan et al., 2015). Unfortunately, without any phenotypic data, we are not able to identify specific traits potentially underlying this selected region. Further studies are needed to answer this question.

In summary, we comprehensively characterized the genetic composition of GLP breed. The phylogenetic analysis shows that GLP is closest to pointer breeds originating from Germany in comparison to all the other breeds included in this study. Through inbreeding analyses, we showed that Dutch GLPs have relatively low inbreeding in comparison to 11 other pointer setter breeds. However, inbreeding varies considerably and is associated with the incidence of thyroid cancer within GLPs, suggesting that inbreeding control is essential for breeding healthy GLPs. We also identified a genomic region under selection on chromosome 30 that might contribute to hunting performance of GLPs, harboring the genes *RYR3*, *FMN1*, and *GREM1*.

## CONFLICT OF INTEREST

The authors declare no conflict of interest.

## Supporting information


Figures S1–S12

## Data Availability

Sequencing data presented in this study are openly available at EMBL‐EBI ENA database with reference number PRJEB43017. SNP array genotype data are available through ArrayExpress (accession number E‐MTAB‐10241).
