# Supplementary material for: Unique genetic signature and selection footprints in Dutch population of German Longhaired Pointer dogs
Source: Anim Genet. 2022 Aug 22;53(6):829–40. doi: 10.1111/age.13253 (PMC9804189; doi:10.1111/age.13253)
Supplement: Supplementary file 1 — Figures S1–S12 [file AGE-53-829-s001.docx]

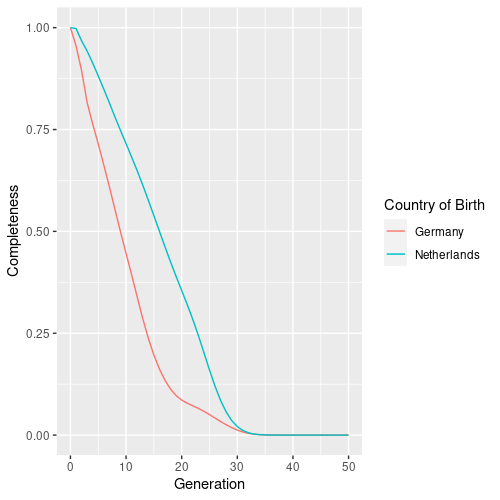


Figure S1. Pedigree completeness of GLPs born in Germany and the Netherlands.


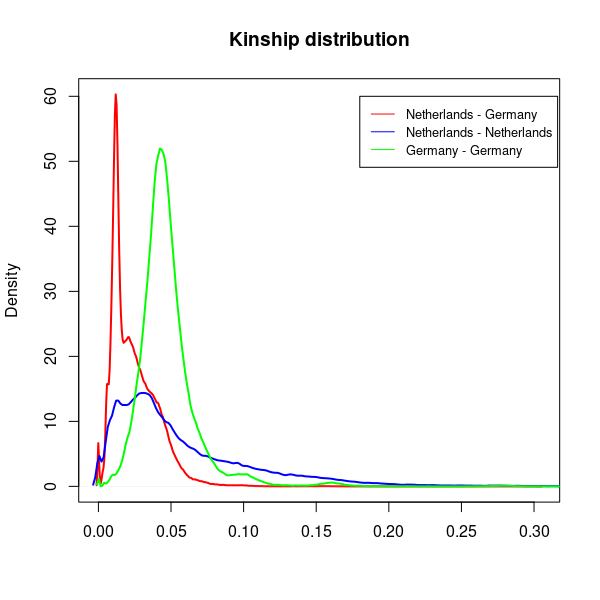


Figure S2. Kinship between GLPs born in the Netherlands and Germany after year 2000.


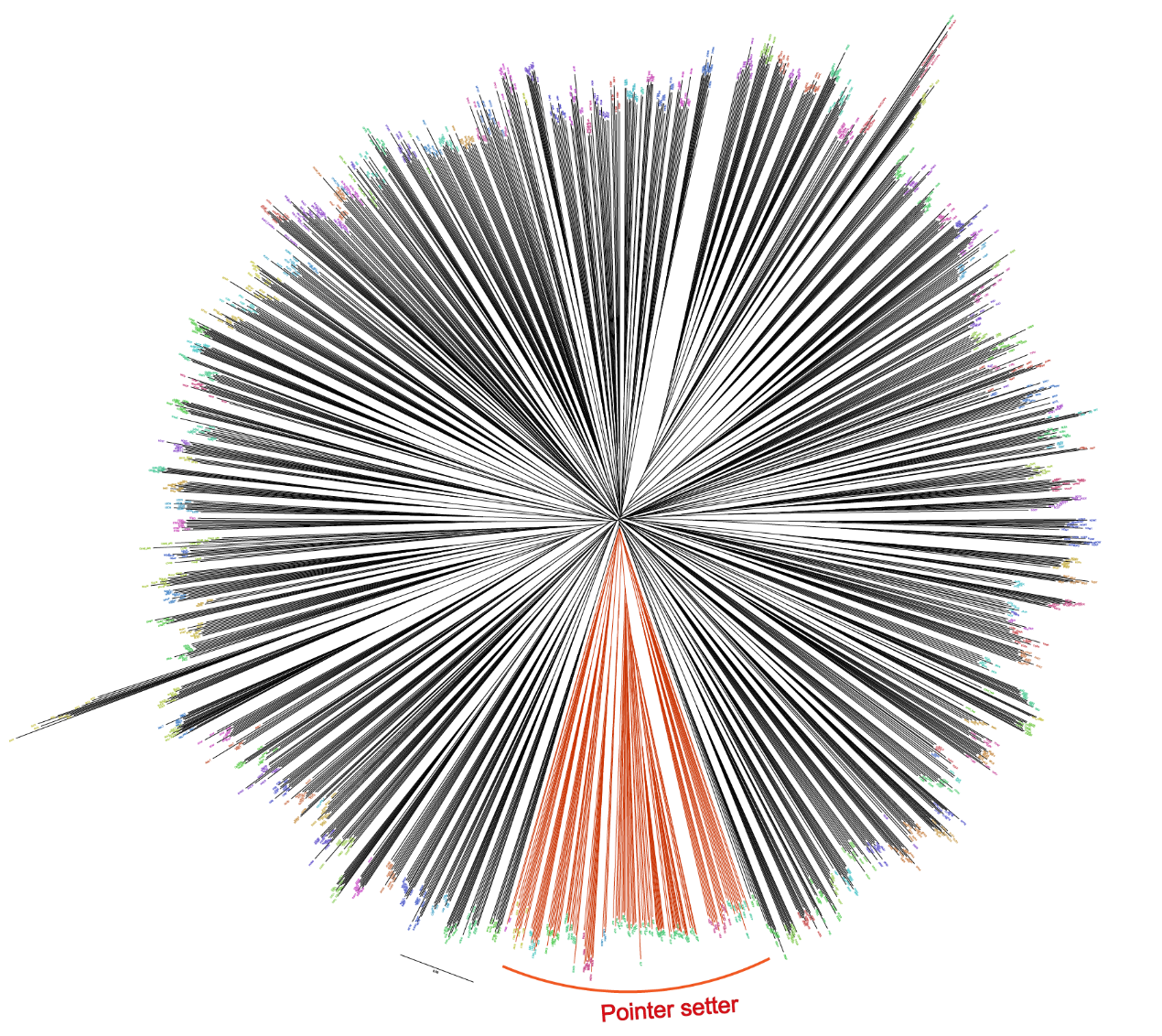


Figure S3. Neighbor-joining phylogenetic tree constructed based on genotype data of 1386 dog from 162 breeds. Highlighted subclade is the pointer setter subclade where German Longhaired Pointers locate on.


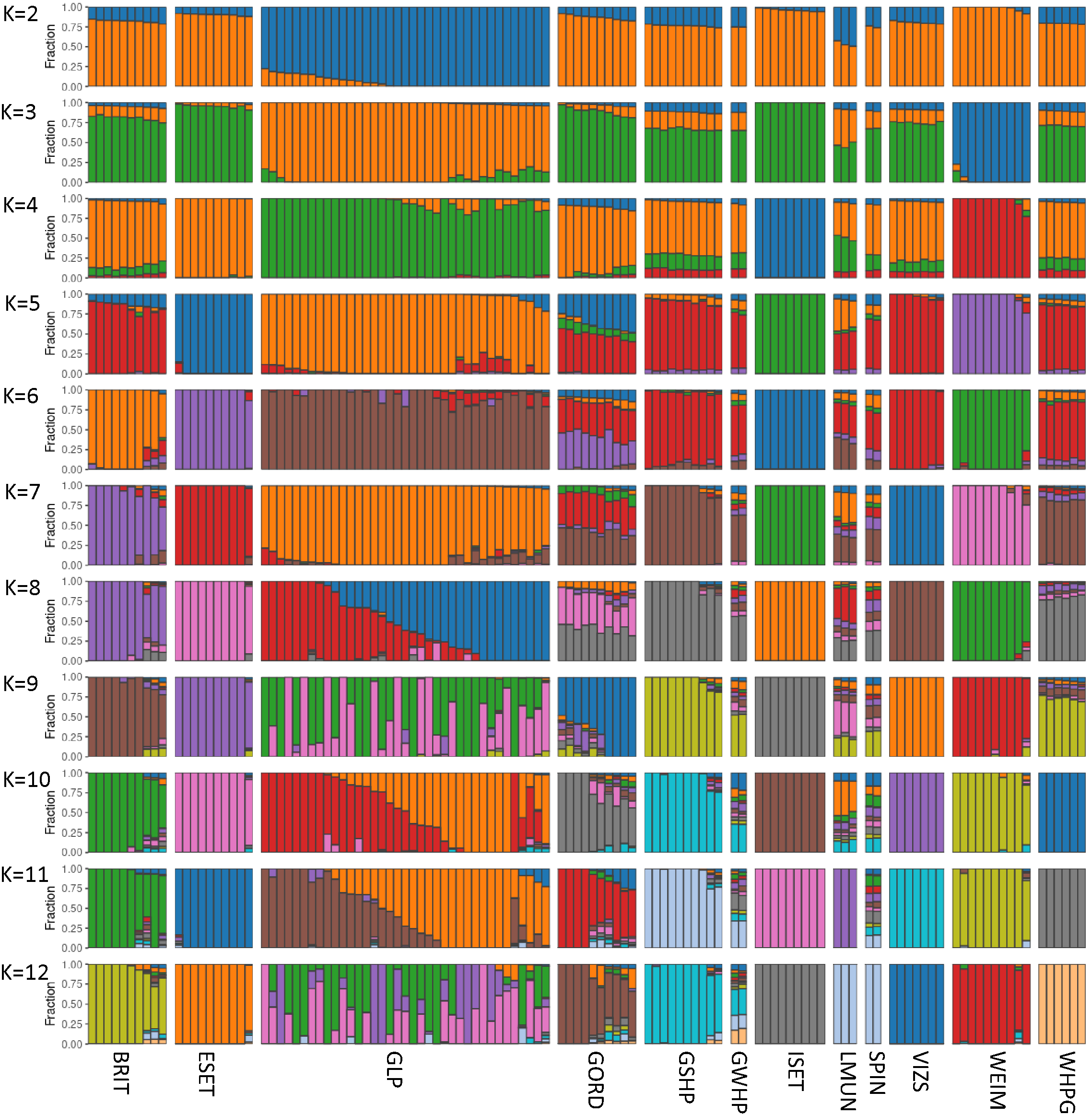


Figure S4. Population structure of the 12 pointer setter breeds estimated by ADMIXTURE with inferred cluster ranging from 2 to 12.


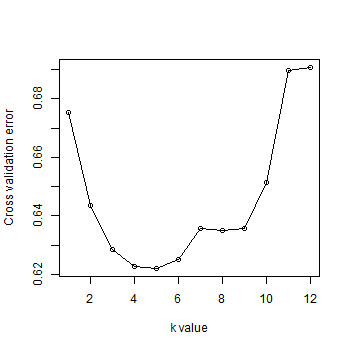


Figure S5. Cross-validation errors for different K values in the ADMIXTURE analysis.


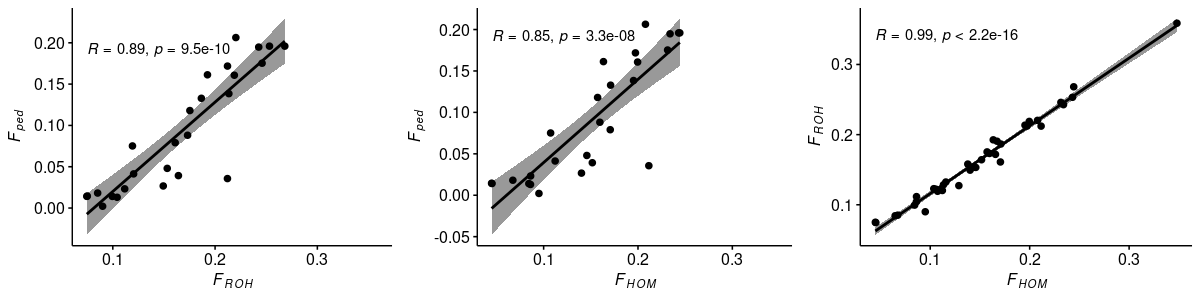


Figure S6. Correlation between Fped, F_ROH_, and F_HOM_ of GLPs.


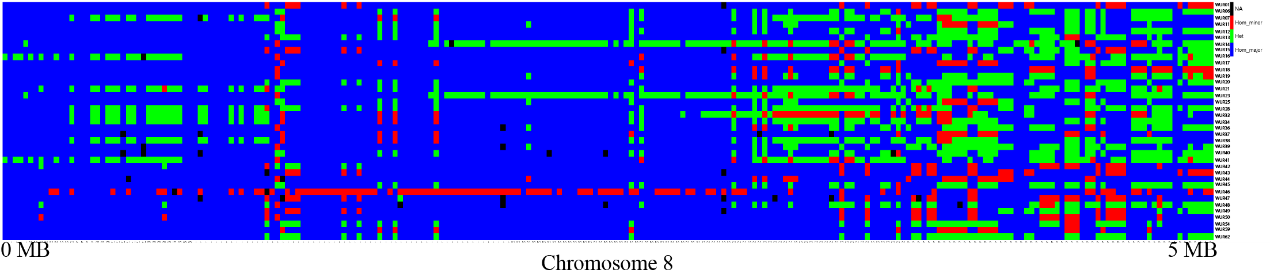


Figure S7. Genotypes of the variants between 0 and 5 Mb on chromosome 8 in 37 GLPs. Colors denote homozygous for major allele (allele), heterozygous (green), homozygous for minor allele (red), and missing genotype (black) .


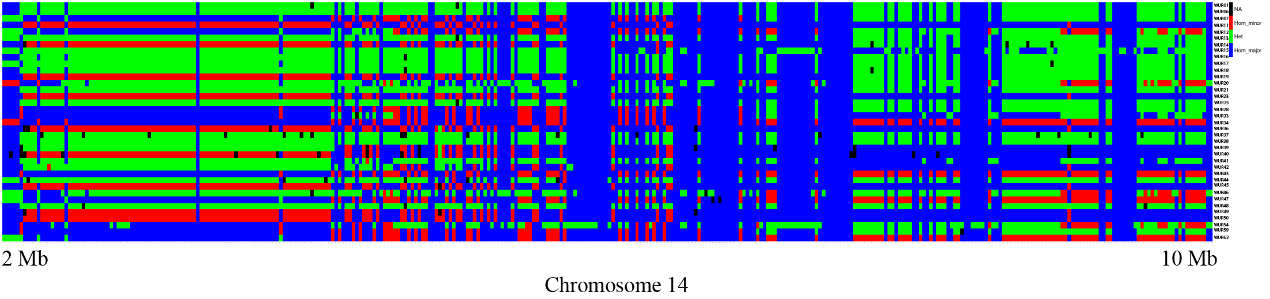


Figure S8. Genotypes of the variants between 2 and 10 Mb on chromosome 14 in 37 GLPs. Colors denote homozygous for major allele (allele), heterozygous (green), homozygous for minor allele (red), and missing genotype (black).


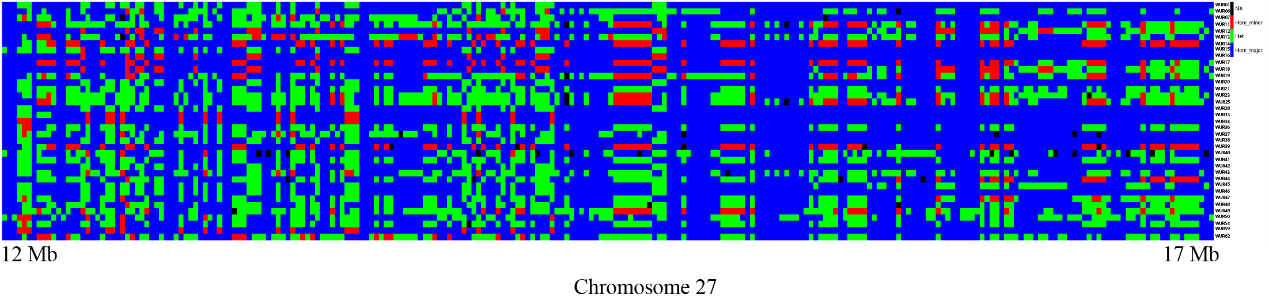


Figure S9. Genotypes of the variants between 12 and 16 Mb on chromosome 27 in 37 GLPs. Colors denote homozygous for major allele (allele), heterozygous (green), homozygous for minor allele (red), and missing genotype (black).


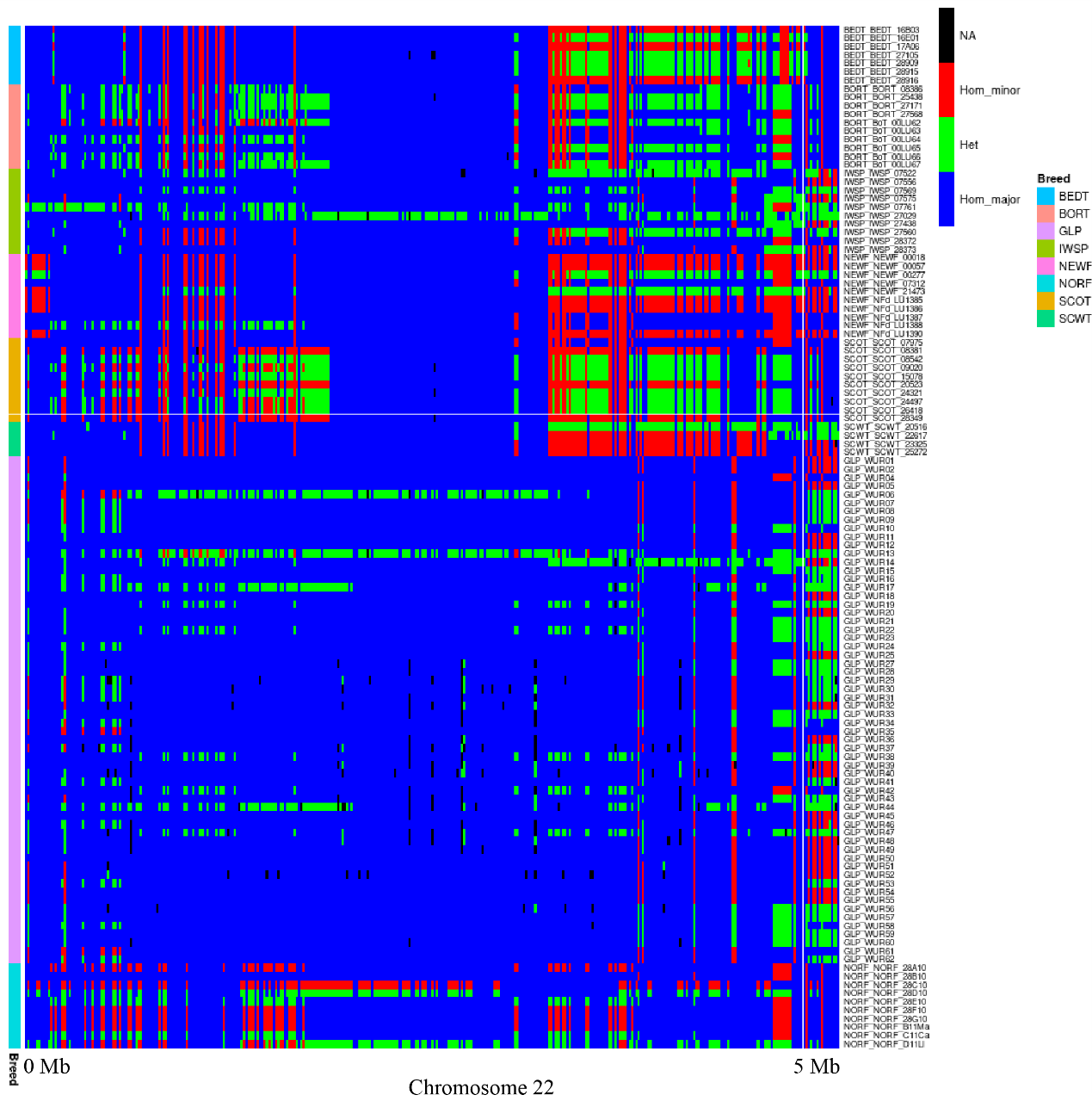


Figure S10. Genotypes of the variants betweean 0 and 5 Mb on chromosome 22 in German Longhaired Pointer (GLP), Bedlington Terrier (BEDT), Border Terrier (BORT), Irish Water Spaniel (IWSP), Newfoundland (NEWF), Norfolk Terrier (NORF), Scottish Terrier (SCOT) and Soft Coated Wheaten Terrier (SCWT). Colors denote homozygous for major allele (allele), heterozygous (green), homozygous for minor allele (red), and missing genotype (black).


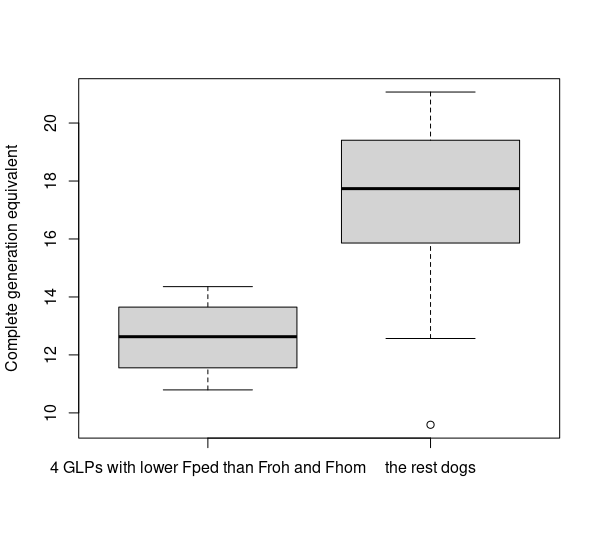


Figure S11. Comparison of complete generation equivalents between 4 GLPs that have lower Fped than genotype-based inbreeding and the rest 33 GLPs.


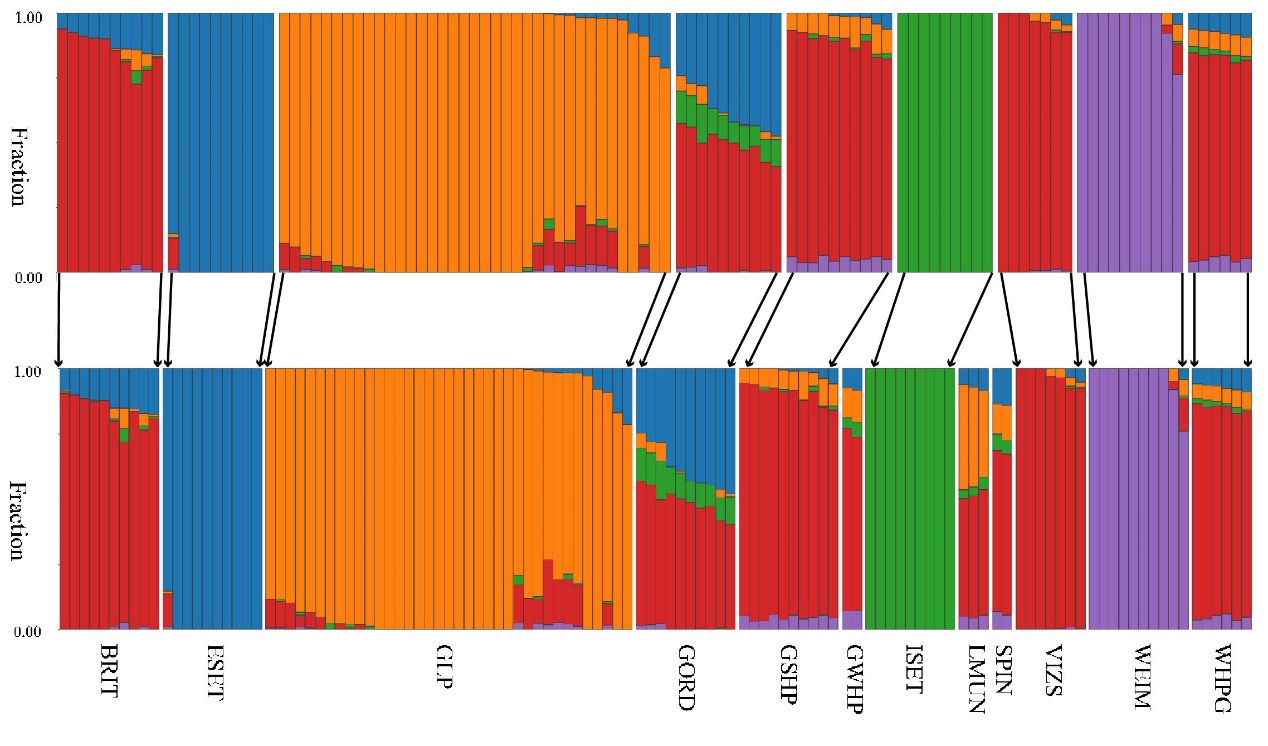


Figure S12. Top panel shows ADMIXTURE analysis result when 3 breeds (GWHP, LMUN, SPIN) with less than 4 samples were removed. Bottom panel shows ADMIXTURE analysis result using all 12 pointer setter breeds. Abbreviations: BRIT – Brittany, ESET – English Setter, GLP – German Longhaired Pointer, GORD – Gordon Setter, GSHP – German Shorthaired Pointer, GWHP – German Wirehaired Pointer, ISET – Irish Setter, LMUN – Large Munsterlander, SPIN – Spinone Italiano, VIZS – Vizsla, WEIM – Weimaraner, WHPG – Wirehaired Pointing Griffon.
